# Reciprocal effects among changes in weight, body image, and other psychological factors during behavioral obesity treatment: a mediation analysis

**DOI:** 10.1186/1479-5868-6-9

**Published:** 2009-02-09

**Authors:** António L Palmeira, David A Markland, Marlene N Silva, Teresa L Branco, Sandra C Martins, Cláudia S Minderico, Paulo N Vieira, José T Barata, Sidónio O Serpa, Luis B Sardinha, Pedro J Teixeira

**Affiliations:** 1 Faculty of Human Kinetics, Technical University of Lisbon, Estrada da Costa, 1495-688, Cruz Quebrada, Portugal; 2 University Lusófona de Humanidades e Tecnologias, Campo Grande, 1749-028, Lisbon, Portugal; 3 School of Sport, Health & Exercise Sciences, Bangor University, George Building, Holyhead Road, Bangor, Gwynedd, UK

## Abstract

**Background:**

Changes in body image and subjective well-being variables (e.g. self-esteem) are often reported as outcomes of obesity treatment. However, they may, in turn, also influence behavioral adherence and success in weight loss. The present study examined associations among obesity treatment-related variables, i.e., change in weight, quality of life, body image, and subjective well-being, exploring their role as both mediators and outcomes, during a behavioral obesity treatment.

**Methods:**

Participants (BMI = 31.1 ± 4.1 kg/m^2^; age = 38.4 ± 6.7 y) were 144 women who attended a 12-month obesity treatment program and a comparison group (n = 49), who received a general health education program. The intervention included regular group meetings promoting lasting behavior changes in physical activity and dietary intake. Body image, quality of life, subjective well-being, and body weight were measured at baseline and treatment's end. Mediation was tested by multiple regression and a resampling approach to measure indirect effects. Treatment group assignment was the independent variable while changes in weight and in psychosocial variables were analyzed alternatively as mediators and as dependent variables.

**Results:**

At 12 months, the intervention group had greater weight loss (-5.6 ± 6.8% vs. -1.2 ± 4.6%, p < .001) and larger decreases in body size dissatisfaction (effect size of 1.08 vs. .41, p < .001) than the comparison group. Significant improvements were observed in both groups for all other psychosocial variables (effect sizes ranging from .31–.75, p < .05). Mediation analysis showed that changes in body image and body weight were concurrently mediators and outcomes of treatment, suggesting reciprocal influences. Weight loss partially mediated the effect of treatment on quality of life and on self-esteem but the reciprocal effect was not observed.

**Conclusion:**

Changes in weight and body image may reciprocally affect each other during the course of behavioral obesity treatment. No evidence of reciprocal relationships was found for the other models under analysis; however, weight changes partially explained the effects of treatment on quality of life and self-esteem. Weight and psychosocial changes co-occur during treatment and will probably influence each other dynamically, in ways not yet adequately understood. Results from this study support the inclusion of intervention contents aimed at improving body image in weight management programs.

## Background

Improving the treatment of overweight and obesity remains a critical challenge [1]. Several health behavior change models, often based on a social-cognitive framework, have been used to design weight management interventions, helping researchers improve treatment contents and conditions aiming at weight loss and maintenance [2]. However, most interventions have only produced modest weight reductions, especially in the long-term [3,4], and social-cognitive variables have shown limited power to predict weight outcomes [5,6]. Other predictors, and possibly alternative explanatory models, are needed to better understand the mechanisms by which successful weight management and other obesity treatment outcomes are more likely to occur [7-10].

Although findings are not entirely consistent, obesity intervention studies report improvements in other outcomes besides weight loss, such as body image, quality of life, self-esteem, and depression [11,12]. Results generally show that psychosocial outcomes are more evident in the long-term, and that they are not always associated with weight loss. For example, improved body image is inconsistently associated with treatment-related weight changes [13,14], whereas quality of life improvements – especially using obesity-specific measures – are more strongly associated with weight change [15]. One recent meta-analysis on the role of subjective well-being in obesity treatment suggested that self-esteem increases are dependent on weight loss, regardless of treatment group, whereas reductions in depression are independent of weight loss, but strongly associated with treatment [11]. Another meta-analysis showed that neither depression nor self-esteem improvements were associated with treatment condition; however, this study did not report associations between these variables and weight change [12]. These reports analyzed psychosocial changes primarily as outcomes of treatment. However, several authors have recommended that psychosocial changes should also be analyzed as mechanisms that can potentially contribute to better weight results, for example by mediating intervention effects on behavioral adherence and weight loss [16,17].

More than a decade ago, Friedman and Brownell recommended a "third generation" of obesity treatment studies, analyzing causal mechanisms and interactions between psychosocial variables and weight change [18]. Underlying their recommendation was the hypothesis that these paths might be intertwined and reflect reciprocal influences, which is coherent with the concept of reciprocal determinism between individual, environment, and behavior, central to Bandura's Social Cognitive Theory [19]. For example, a treatment might be effective partially because it increases psychological well-being, which in turn helps produce weight loss while, concurrently, weight changes might have also helped produce improved psychological well-being (e.g., body image or self-esteem). This phenomenon could be studied by reciprocal effects analysis, which is an extension of the traditional mediating model approach [2] and echoes the reciprocal effects model (REM) suggested by Marsh and colleagues in educational psychology research [20].

To our knowledge, reciprocal effects analyses have never been explored for weight loss and weight-related behaviors. Therefore, in the context of a 1-year behavior weight management program with adult women, the present study was designed to: a) analyze associations among treatment-related outcomes – changes in weight, quality of life, body image, and subjective well-being (i.e., self-esteem and depressive symptoms); and b) analyze the potential role of each of these variables as both mediators and outcomes, i.e., study reciprocal effects among these variables during (and as a result of) the treatment.

We predicted that intervention-related changes in body image and subjective well-being would both mediate and be mediated by weight change (i.e., reciprocal effects will be present), whereas quality of life would be mediated by weight change but not the reverse. Body image and subjective well-being variables have sometimes been associated with weight loss and their improvement during treatment is a consistent finding [13,14,17]. Therefore, they may be playing a double role as both mediators and outcomes, i.e., influencing and being influenced by weight loss. Conversely, obesity-specific measures of quality of life are consistently associated with both treatment participation and weight loss [11,12]. However, since these measures lead participants to reflect on quality of life as a consequence of their weight (e.g., 'because of my weight I am less productive than I could be'), we expect a one-way mediation to be present, from weight loss to improved quality of life (but not in the opposite direction).

## Methods

### Participants

Female participants were recruited from the community for two successive long-term weight management programs, which had very similar contents and intervention approaches, through newspaper ads, a website, email messages, and announcement flyers. Participants were required to be older than 24 years, pre-menopausal, not pregnant, have a BMI between 25 and 40 kg/m^2^, and be free from major disease, to be eligible for the studies. For the present analyses, we used only participants who had completed 12-month assessments, comprising 193 women in total (BMI = 31.1 ± 4.1 kg/m^2^; Age = 38.4 ± 6.7 y). No differences were observed between 32 non-completers (14.3% attrition) and the 193 completers in the baseline assessments of the variables (p > .10). The intervention group (pooled from both studies, described below) included 144 participants. The comparison group had 49 participants, who did not receive a weight loss program. These participants derived from only one of the two studies, because in one of the designs all participants engaged in weight loss programs (with different levels of intervention). They received a general health education program comprising of 15 sessions covering topics such as stress management, general healthy eating, and cardiovascular risk reduction, among others. The intervention group was slightly older than the comparison group (39.0 ± 6.6 vs 36.6 ± 6.8 y, p = .032) and displayed lower self-esteem scores at baseline (p = .001), but there were no differences between groups with regard to weight, BMI, the proportion of participants who were obese (see Table 1), or other psychosocial variable. All participants agreed to refrain from participating in any other weight loss program and signed a written informed consent prior to participation in the study. The Faculty of Human Kinetic's Human Subjects Institutional Review Board approved the study.

**Table 1:** Baseline demographic and anthropometric characteristics of the participants

	Intervention (n = 144)	Comparison (n = 49)
	M ± SD	M ± SD
Weight (kg)	80.7 ± 12.2	79.7 ± 12.6
BMI (kg/m^2^)	31.2 ± 4.2	30.7 ± 3.8
Age (years)	39.0 ± 6.6	36.6 ± 6.8
	
	Percentage	Percentage
% Obese	57.1	56.3

Note: No differences between groups, except for age (p = .032)

### Intervention

The intervention group sessions, which lasted for about 1 year, included exercise, nutrition, and behavior modification topics and were loosely based on the LEARN weight management program [21]. In one of the programs women (n = 81) met weekly with the intervention team for 4 months, then monthly for the remaining period. In the other, participants (n = 63) met weekly or every two weeks throughout the 12 months. Participants met with the intervention team in groups of approximately 30, for 120–150 min per session. The interventions included educational content and practical applications in the areas of physical activity and exercise, diet and eating behavior, behavior modification, and have been partially described before [5,22]. Physical activity topics included learning the energy cost associated with typical activities, increasing daily walking and lifestyle physical activity, planning and implementing a structured exercise plan, and choosing the right type of exercise, among many others. Examples of covered nutrition topics were learning the caloric, fat, and fiber content and the energy density of common foods, the role of breakfast and meal frequency for weight control, reducing portion size, and preventing binge and emotional eating. Cognitive and behavioral skills including self-monitoring, self-efficacy enhancement, dealing with lapses and relapses, enhancing body image, using contingency management strategies, and eliciting social support were also part of the curriculum. Sessions were conducted by the same team composed of Doctoral and Masters level exercise physiologists, psychologists, and dieticians. For each group, a group leader was selected from the intervention team to be present in all meetings. Participants were informed that weight reduction should be understood as a long-term goal, and that a 5–10% weight loss was an appropriate goal to be sought at the end of the program.

### Instruments

#### Psychosocial Variables

Data were collected in two periods: a) baseline, corresponding to the pre-treatment scores; and b) at 12 months, which corresponded to the end of the treatment. The instruments were Portuguese validated versions of some of the most widely used psychosocial instruments in obesity research.

#### Body Image

Body Image was evaluated by two questionnaires commonly used to measure this construct, which were analyzed separately to consider body image's multidimensional nature [23]. Body size dissatisfaction (BSD) was measured with the Body Image Assessment questionnaire – BIA [24,25], which consists of nine silhouettes of increasing size, from which participants are asked to select the figures corresponding to their current (i.e., perceived actual body size) and their ideal body size. Body size dissatisfaction was calculated by subtracting the score for perceived body size from the ideal body size rating. Lower values indicate higher levels of body size dissatisfaction. The Body Shape Questionnaire – BSQ [25,26], a 34-item instrument scored on a 6-point Likert scale, was used to measure affective, cognitive, and behavioral dimensions of body image, especially regarding the experience of, and preoccupation with "being fat". The total score was used (α = .95), where higher values represent greater preoccupation with body shape (range 34–204).

#### Quality of Life

Obesity-specific quality of life was assessed using the Impact of Weight on Quality of Life – Lite – IWQOL-L [27,28], a 31-item questionnaire scored on a 5-point Likert scale. This measure results in five subscales and a total score in which higher values represent greater quality of life (range 0–100). Only the total score was used in the present study (α = .97).

#### Subjective Well-Being

Self-esteem was assessed with the Rosenberg Self-Esteem Scale – RSES [29,30], composed of 10 items answered on a 4-point Likert scale. Higher scores on the RSES represent greater self-esteem (α = .76, range 10–40). Depressive symptoms were evaluated with the Beck Depression Inventory – BDI [31,32], a 21-item inventory measuring several symptoms of depression. It is scored on a 4-point scale and results in a total depression score (α = .91), where higher scores represent greater levels of depressive symptoms (range 0–63).

#### Body Weight

Body weight was lab-measured first thing in the morning in fasting conditions, with participants in light clothing, with a standardized procedure (average of three measures was used) at both baseline and treatment's end (12 months), using an electronic scale (SECA model 770, Hamburg, Germany).

### Statistical Procedures

A mixed models ANOVA (time × group) was used to analyze the impact of the program on weight and psychosocial variables. Pearson correlation was used to examine associations between changes in weight and the psychosocial constructs. For correlational analysis, variables were expressed by the residuals of the 12-month value regressed on the baseline score. Using such residualized change scores is recommended as it creates a value that is orthogonal to the pre-treatment value(s) and represents a preferable measure of change, when compared with the pre-post subtraction procedure [33]. For ease of interpretation of the correlational results, body image and depression scores were reversed, so that for all variables in the study higher scores always represent a more positive outcome.

To test the mediation models we used the procedures described by Preacher and Hayes [34], which use multiple linear regression analysis. Treatment vs. comparison was the independent variable, while changes in body weight, body image, quality of life, and subjective well-being played were tested both as mediators and dependent variables, consistent with the reciprocal effects model under analysis. Therefore, we had five reciprocal effect models, for a total of 10 regressions. For example, the two regression models for quality of life had the following structure: a) Independent variable: treatment group; Dependent variable: weight changes; Mediator: quality of life changes; and b) Independent variable: treatment group; Dependent variable: quality of life changes; Mediator: weight changes.

Reciprocal effects were considered to be present when significant mediation occurred in both regression models for a given construct. In the previous example, this would occur if both quality of life mediated treatment effects on weight and if weight changes mediated treatment effects on quality of life. This inference was made either with complete or partial mediation by the proposed mediators.

Preacher and Hayes [34] have recently provided a SPSS macro for the analyses of the causal steps criteria for mediation forwarded by Baron and Kenny [35] including Sobel tests, and also bootstrapped resampling results, for the specific indirect (or mediated) effects. We will present the resampling procedure (5000 bootstrap samples), via the Bias Corrected and Accelerated (BCa) estimates and 95% confidence intervals to present the indirect effects' significance. The BCa confidence intervals are considered by Preacher and Hayes [34] superior to the normal theory Sobel tests as they require no distributional assumptions and are less likely to lead to a Type I error. If the BCa 95% confidence interval does not include zero we can conclude there was a significant indirect effect (at alpha = .05). Collinearity was tested, resulting in variance inflation factors (1.10 – 1.87) and tolerances (.53 – .91) within the limits accepted for regression analysis [33]. Homoscedasticity and linearity were observed through the analysis of residual scatterplots, revealing no problems.

## Results

The analysis of the impact of the program on the design groups is presented in Table 2.

**Table 2:** Means, standard deviations, effect sizes and mixed model ANOVA to analyze the impact of the program on the intervention (n = 144) vs comparison (n = 49) groups

	Baseline	12 Months		Time × Group	
	
*Variables*	M ± SD	M ± SD	ES	F	p
**Weight (kg)**					
Comparison	79.7 ± 12.6	78.7 ± 12.3	-0.09	16.79	<.001
Intervention	80.7 ± 12.2	76.1 ± 12.1	-0.38		
**Body Image**					
Body size dissatisfaction					
Comparison	2.5 ± 0.8	2.2 ± 0.8	-0.41	12.11	<.001
Intervention	2.4 ± 0.7	1.6 ± 0.7	-1.08		
Body shape concerns					
Comparison	99.0 ± 22.1	85.2 ± 24.2	-0.60	2.45	0.120
Intervention	96.5 ± 27.7	76.1 ± 26.4	-0.75		
**Quality of Life**					
Weight-Related QOL					
Comparison	73.8 ± 13.9	82.2 ± 12.2	0.64	0.22	0.639
Intervention	79.5 ± 13.5	87.0 ± 10.3	0.63		
**Subjective Well-Being**					
Self-esteem					
Comparison	33.0 ± 4.1	34.3 ± 4.5	0.31	2.18	0.140
Intervention	30.5 ± 4.4	32.8 ± 4.4	0.52		
Depression					
Comparison	6.8 ± 4.6	3.8 ± 3.5	-0.75	0.50	0.480
Intervention	6.6 ± 4.7	4.4 ± 4.4	-0.49		

Note: ES – effect size. QOL – Quality of Life.

Weight loss was smaller in the comparison group (-1.2 ± 4.6%, p = .060) when compared to the intervention group (-5.6 ± 6.8%, p < .001). Body size dissatisfaction was reduced more effectively in the intervention group. All other variables improved during the program in both groups (p < .05), although treatment-related differences between groups were non-significant (see Table 2).

Table 3 shows the intercorrelations among weight and psychosocial changes. Weight change was associated with changes in all psychosocial variables except depression. Among psychosocial variables, only self-esteem and body size dissatisfaction were not positively correlated.

**Table 3:** Internal consistency (Alpha) for psychosocial variables and intercorrelations among weight and psychosocial changes (n = 193).

	Alpha	Weight Change	*1*	*2*	*3*	*4*
**Body Image Changes**						
1 Body size dissatisfaction		-.49 ***				
2 Body shape concerns	.95	-.41 ***	.33 ***			
**Quality of Life Changes**						
3 Weight-Related QOL	.97	-.45 ***	.37 ***	.60 ***		
**Subjective Well-Being Changes**						
4 Self-esteem	.76	-.21 **	.08	.37 ***	.41 ***	
5 Depression	.91	-.14	.18 *	.33 ***	.36 ***	.20 *

Note: *p < .05, **p < .01; ***p < .001. QOL – Quality of Life. The Body Size Dissatisfaction does not have an internal consistency value.

**Figure 1:**
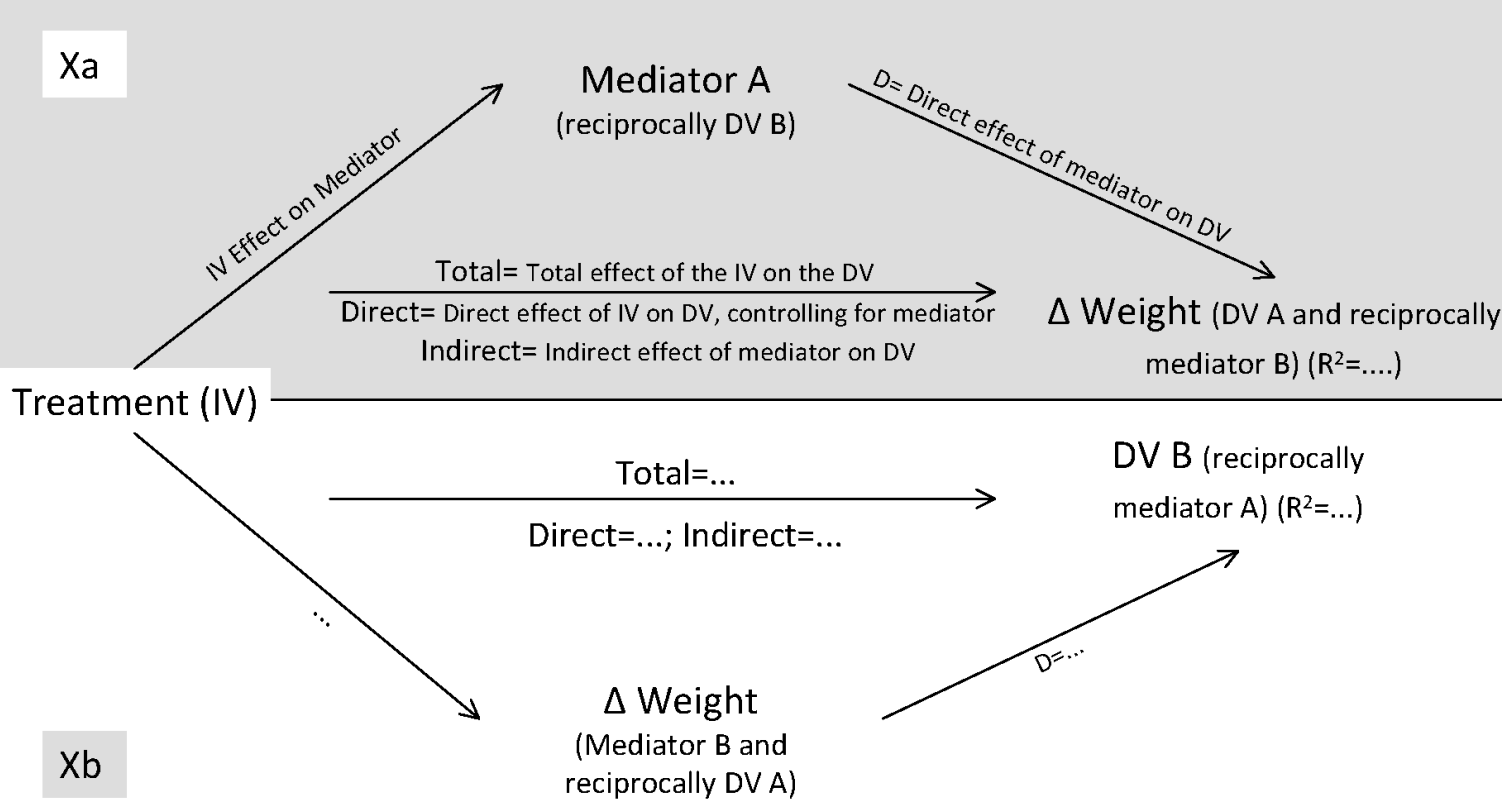
**Indications to read the results of the reciprocal mediation-result figures**. **Note for Figure 1**. All values are standardized coefficients (except for the R^2^); IV – Independent Variable; DV – Dependent Variable.

**Figure 2:**
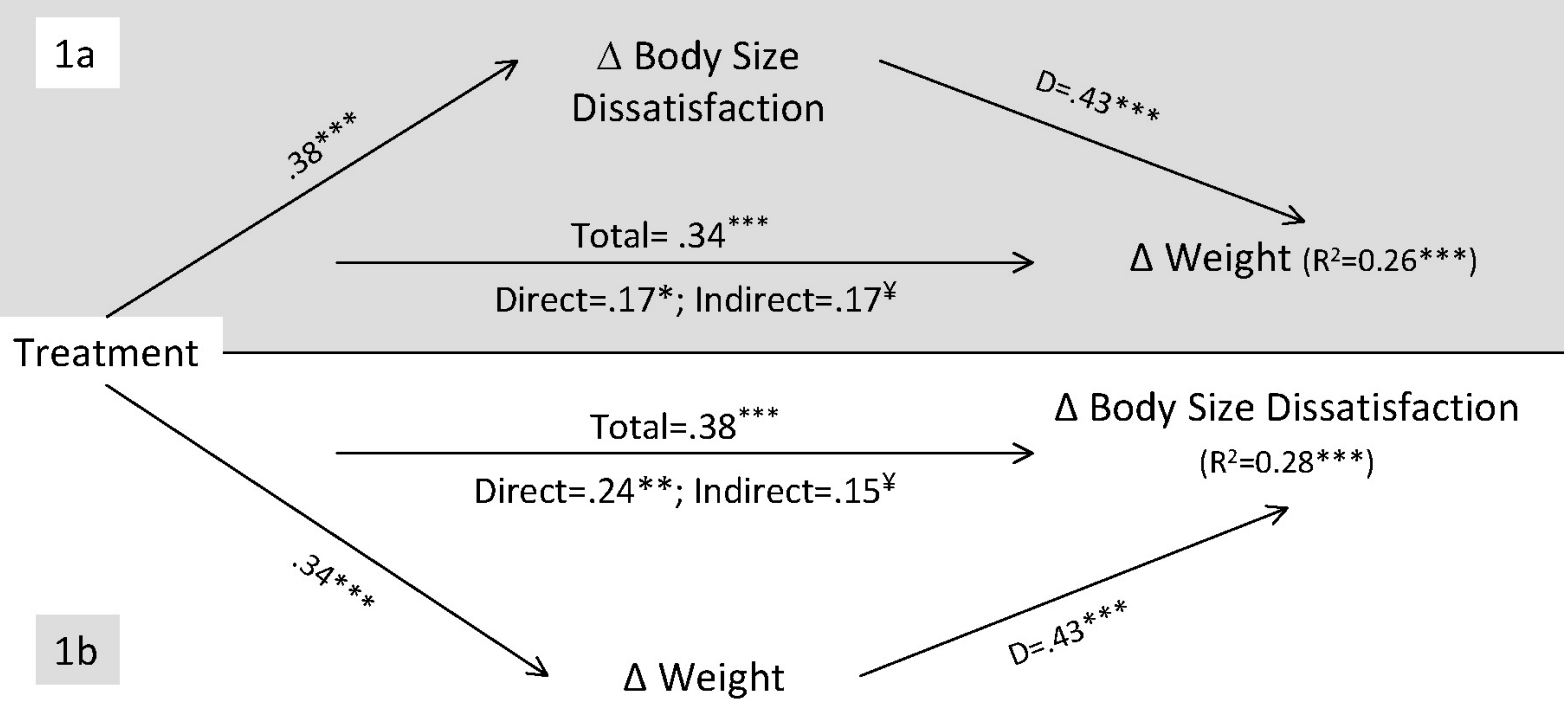
**Mediation analysis for the reciprocal change effects between weight and body size dissatisfaction**. **Note for Figure 2**. See note for figure 1 for more information. *p < .05; **p < .01; ***p < .001. ¥ – The 95% CI of the Bias and Corrected and Accelerated estimate indicate a significant indirect effect.

**Figure 3:**
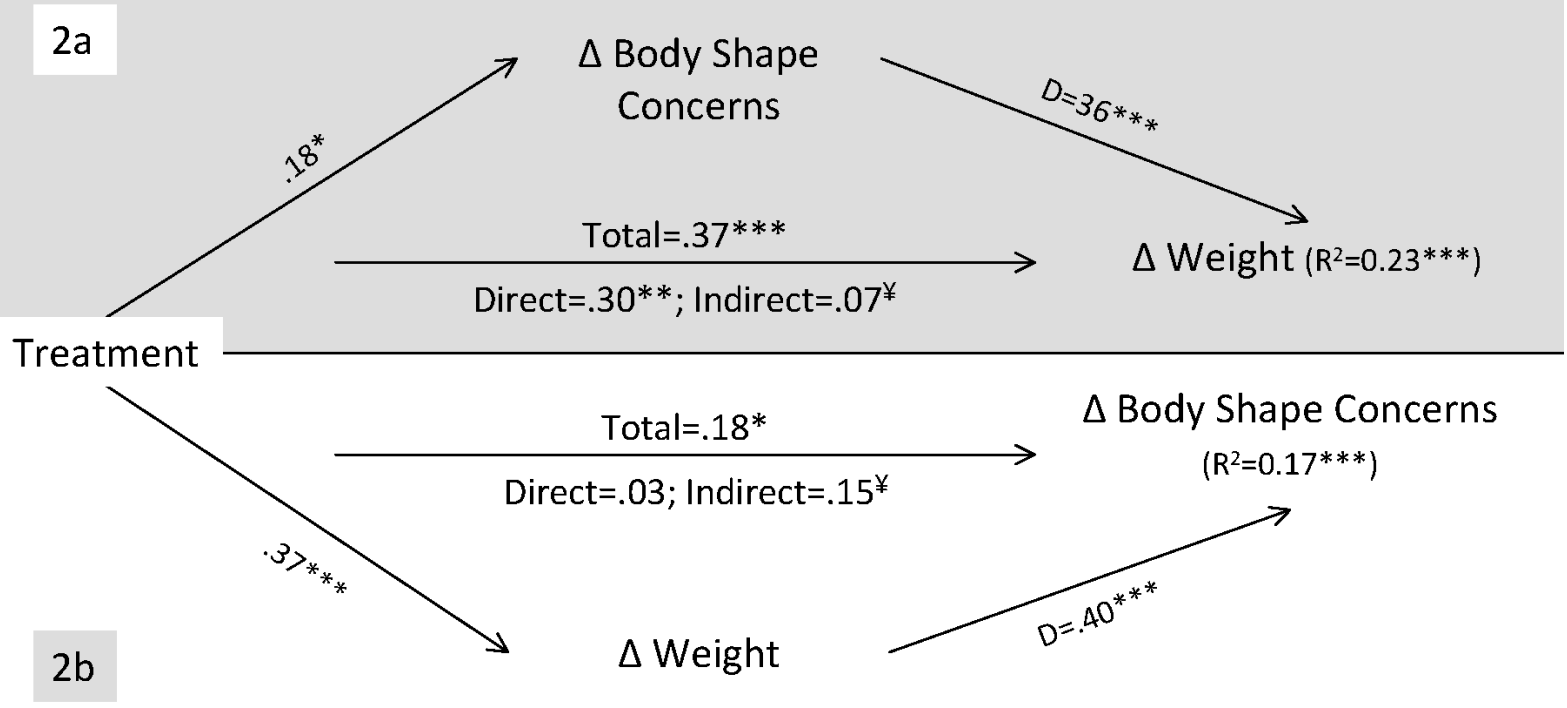
**Mediation analysis for reciprocal change effects between weight and body shape concerns**. **Note for Figure 3**. See note for figure 1 for more information. *p < .05; **p < .01; ***p < .001. ¥ – The 95% CI of the Bias and Corrected and Accelerated estimate indicate a significant indirect effect.

**Figure 4:**
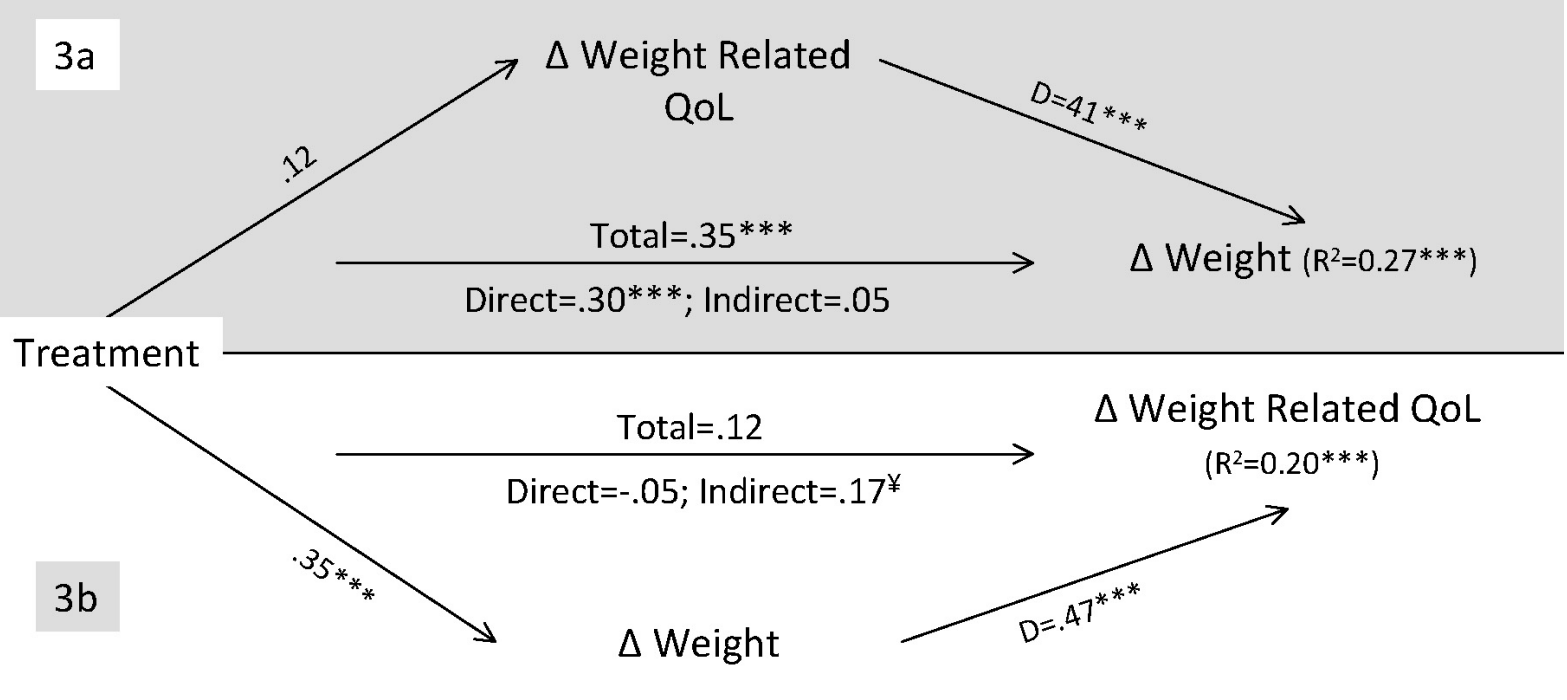
**Mediation analysis for reciprocal change effects between weight and weight related quality of life**. **Note for Figure 4**. See note for figure 1 for more information. *p < .05; **p < .01; ***p < .001. ¥ – The 95% CI of the Bias and Corrected and Accelerated estimate indicate a significant indirect effect. QOL – Quality of Life.

**Figure 5:**
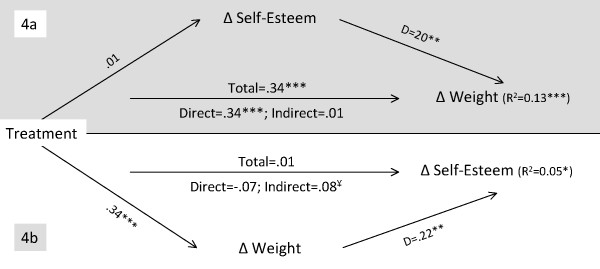
**Mediation analysis for reciprocal change effects between weight and self-esteem**. **Note for Figure 5**. See note for figure 1 for more information. *p < .05; **p < .01; ***p < .001. ¥ – The 95% CI of the Bias and Corrected and Accelerated estimate indicate a significant indirect effect.

The results of the reciprocal mediation analysis are presented next. Figure 1 contains a detailed description of each relationship in these models (useful to interpret Figures 2 to 5). The top model (Xa) shows the mediation analysis for the prediction of weight change using mediator A as the mediator. The lower model (Xb) represents the reciprocal mediation analysis, i.e., the prediction of the dependent variable B (reciprocal mediator A), using weight change (reciprocally dependent variable A) as mediator. We have not included socio-demographic variables as covariates because in preliminary analyses they were not related to weight or psychosocial outcomes.

For example, in the first analysis for the reciprocal hypothesis in body size dissatisfaction (figure 2), the top model (1a) shows the mediation analysis for the prediction of weight change using body size dissatisfaction change as mediator. The lower model (1b) represents the reciprocal mediation analysis, i.e., the prediction of body size dissatisfaction changes using weight change as mediator. The same approach will be used for the presentation of the remaining reciprocal mediation models.

The body size dissatisfaction mediation model (Figure 2, 1a), explained 26% of the weight change variance (F(2,164) = 29.43, p < .001). Total (p < .001), direct (p < .05), and indirect (95% BCa CI of 0.09 to 0.29) effects were significant. The weight change mediation model (Figure 2, 1b), explained 28% of body size dissatisfaction outcomes (F(2,164) = 32.21, p < .001). As in model 1a, all effects were significant, with total (p < .001), direct (p < .01) and indirect effects (95% BCa CI of 0.07 to 0.24) significantly influencing body size dissatisfaction changes. Results are consistent with the presence of a reciprocal effect between changes in weight and changes in body size dissatisfaction during (and as a result of) the intervention. As treatment reduced body size dissatisfaction, which in turn affected weight, weight loss also concurrently helped increase body satisfaction (see also Table 4 for a summary). Both models suggest the presence of partial mediation, since the direct effect, although reduced as evidenced by the significant indirect effects, remained significant when controlling for the mediator. Therefore, decreases in body size dissatisfaction during the program appeared to be one mechanism by which treatment affected body weight, while treatment-related weight changes affected body size dissatisfaction, albeit to a slightly lesser extent.

**Table 4:** Summary of the mediation analysis and support for the reciprocal effects model

**Model**	**Meadiator**	**Outcome**	**Classification**	**Notes**
*Body Size Dissatisfaction*				

1a	Δ BSD	Δ Weight	Partially mediates Δ Weight	Partial support for REM. Slightly stronger effects of changes in body dissatisfaction on weight changes than the opposite model.
1b	Δ Weight	Δ BSD	Partially mediates Δ BSD	

*Body Shape Concerns*				

2a	Δ BSQ	Δ Weight	Partially mediates Δ Weight	Partial support for REM. Weight loss mediation was stronger on body shape concerns than the opposite model.
2b	Δ Weight	Δ BSQ	Fully mediates Δ BSQ	

*Weight-Related Quality of Life*				

3a	Δ WR-QoL	Δ Weight	Irrelevant to Δ Weight	No support for REM. Weight loss has an indirect effect on quality of life improvements.
3b	Δ Weight	Δ WR-QoL	Indirect effect on Δ WR-QoL	

*Self-esteem Change*				

4a	Δ Self-esteem	Δ Weight	Irrelevant to Δ Weight	No support for REM. Weight loss has an indirect effect on self-esteem improvements.
4b	Δ Weight	Δ Self-esteem	Indirect effect on Δ Self-esteem	

*Depression Change*				

5a	Δ Depression	Δ Weight	Irrelevant to Δ Weight	No support for REM.
5b	Δ Weight	Δ Depression	Irrelevant to Δ Depression	

Note: Δ – Difference from baseline to program's end; REM – Reciprocal effect model; BSD – Body Size Dissatisfaction; BSQ – Body Shape Concerns; WR-QoL – Weight-Related Quality of Life.

The body shape concerns mediation model (Figure 3, 2a) explained 23% of weight change (F(2,169) = 25.75, p < .001). The total (p < .001), direct (p < .01) and indirect effects (95% BCa CI of 0.01 to 0.14) were significant, therefore changes in body shape concerns partially mediated total treatment effects. The weight change mediation model (Figure 3, 2b) explained 17% of the variance in body shape concerns (F(3,169) = 16.83, p < .001). The indirect effects of weight change exerted a complete mediation of the effects of treatment on body shape (95% BCa CI of 0.08 to 0.23), since the significant total effect was reduced to a non-significant direct effect when controlling for the mediator. Results suggest reciprocal effects between changes in weight and body shape concerns during, and as a result of treatment. In other words, treatment reduced body shape concerns leading to weight loss, while reductions in weight were also associated with reductions in body shape concerns (see also Table 4). Weight loss appears to be a strong mechanism by which the intervention reduced concerns with body shape and feelings of being too fat.

The weight-related quality of life mediation model (Figure 4, 3a) explained 27% of weight change (F(2,172) = 32.08, p < .001). Treatment total and direct effects were significant, while the indirect effect was non-significant (i.e., no mediation). The weight change mediation model (Figure 4, 3b) explained 20% of quality of life treatment-related outcomes (F(2,172) = 21.75, p < .001). Contrary to the previous model, treatment effects were non-significant, whereas the indirect effect was significant (95% BCa CI of 0.10 to 0.26). Results do not support the presence of reciprocal effects. Weight change had an indirect effect of treatment-related changes in quality of life (model 3b) consistent with treatment producing weight loss which in turn positively affects weight-related quality of life. This situation occurred despite no main effects being detected for treatment impact on quality of life (intervention vs comparison), as neither the total nor the direct effects were significant. Thus, only when treatment affected weight did the intervention produce better weight-related quality of life. However, the alternative model showed that changes in weight-related quality of life did not play a role in the treatment effect on weight change.

The self-esteem mediation model (Figure 5, 4a) explained 13% the variance in weight change (F(2,183) = 13.79, p < .001). Treatment total and direct effects were significant and the indirect effects were non-significant. Conversely, the weight change mediation model (Figure 5, 4b) explained 5% of the variance in change in self-esteem (F(2,183) = 4.39, p = .014). Treatment effects were non-significant, whereas indirect effects were significant (95% BCa CI of 0.03 to 0.15). Results indicate the absence of reciprocal influences between changes in self-esteem and changes in weight during treatment (see also Table 4). These results are very similar to the weight-related quality of life model; only when treatment produced weight loss did the intervention improve self-esteem, since neither total nor direct effects were significant. Therefore, weight change had an indirect effect on the treatment related changes in self-esteem. Nevertheless, the total variance in the dependent variable explained by this model was very small (5%).

The depression mediation model explained 8% of weight change (F(2,125) = 5.45, p = .005). Total treatment effects were significant, whereas the indirect effect was non-significant. The weight change mediation model did not significantly predict depression (F(2,125) = 1.60, p = .206). These models were less predictive as a whole and do not support the reciprocal hypothesis (results not shown).

## Discussion

This study examined the associations among obesity treatment-related variables – i.e., change in weight, quality of life, body image, and subjective well-being -, exploring their potential role as both mediators and outcomes and using a novel analysis approach. We found evidence to suggest that changes in weight and body image may reciprocally affect each other during obesity treatment. A reduction in body size dissatisfaction mediated the treatment effect on weight. The opposite effect (weight loss mediating less body dissatisfaction), although weaker, was also significant suggesting a reciprocal effect between the two variables. Conversely, change in body shape concerns was more dependent on weight outcomes. For the other psychosocial variables, despite no evidence of reciprocal relationships, we observed that weight change partially mediated the effect of treatment on both quality of life and self-esteem, in the expected direction (i.e., more weight loss as a result of treatment resulting in improved psychological outcomes).

The results for body size dissatisfaction suggest that this might have been one of several mechanisms by which the behavioral treatment influenced weight change. This measure of body dissatisfaction assesses self-ideal discrepancy, which can be affected by i) actual change in current body size (or the perception of it); ii) change in ideal body size, for instance, increasing acceptance of a larger than ideal shape/size; or iii) both [24]. The current results seem to point to the last hypothesis, considering the reciprocal effects observed between body size dissatisfaction and weight changes. Taking into account the positive social evaluation of a thin(ner) body, a norm which is internalized by so many people, especially women [16], it is easy to accept that weight loss would mediate improvements in body image during treatment. But could improvements in body image also (i.e. reciprocally) contribute to weight loss, as the present analysis suggests? In other words, might a decreased self-ideal discrepancy about one's body be causally related to improved adherence to the behaviors that lead to weight reduction?

This possibility has been suggested before by Baker and Brownell [36], who proposed that improvements in body image can lead to more adaptive eating and exercise behaviors. Also, Heinberg et al. [37] indicated that there may be an inverted U-shaped relationship between body image dissatisfaction and motivation to lose weight, suggesting that participants who maintain large discrepancies between their perceived actual and ideal body shapes may be caught in a cycle of negative psychological processes (e.g., negative self-talk, rumination, hopelessness) that are debilitating and inhibit change. Furthermore, the lack of progress towards their idealized body size should undermine expectations, possibly resulting in motivational impairments and maladjusted eating behavior patterns [38,39]. Recently, two studies reported mechanisms that might explain these processes regarding physical activity adherence [40,41]. In one of these studies, results showed that higher body size discrepancies were significantly associated with less relative autonomy for exercise in female adolescents [41]. The authors suggested that negative body image leads to less autonomous motivations to exercise, perhaps by increasing the felt pressure to conform to social norms, which in turn inhibits exercise engagement. In the other study [40], with adult females, body size discrepancies exerted a negative influence on physical activity through decreases in the feelings that exercise is a valued and enjoyable activity. We suggest that the body image improvement contents of the treatment, which focused on the development of internal instead of externally or social driven values, could also have helped reduce perceived social pressures and directed the participants towards self-investment and self-acceptance, promoting the development of more autonomous motivations towards behavior change [42].

The second model that dealt with body image showed that weight loss completely mediated treatment effects on body shape concerns, showing that this facet of body image was more dependent on actual weight changes than body size dissatisfaction. Body shape concerns represent distressing preoccupations about weight and shape, embarrassment in public, avoidance of activity (or exposure of the body) due to self-consciousness, and excessive feelings of fatness after eating [26]. Several items in the Body Shape Questionnaire target aspects which seem directly dependent on actual body size and fatness ("When in company have you worried about taking up too much room, e.g., sitting on a sofa or a bus seat?", "Have you avoided running because your flesh might wobble?"). As such, treatment-related increases in body acceptance or changes in what constitutes an ideal body size might not impact this measure of body image as much as they influenced body size dissatisfaction.

In the quality of life models, we observed that only when treatment produced weight loss did the intervention result in improved weight-related quality of life. These results extend previous findings [12,43], where the impact of weight on quality of life was associated with both treatment participation and weight loss. Furthermore, the IWQOL-lite questionnaire is a weight-specific instrument, all of its items starting with the sentence "Because of my weight...". As a result, it is expected that weight change would mediate outcomes in this variable. Several previous studies have shown the IWQOL-lite scores to be strongly correlated with both BMI or body weight change [27,28]. It was interesting to note that despite no significant direct effects of treatment on quality of life, the treatment influence on weight (i.e., the interactive effects of treatment group and weight changes) appeared to predict changes in quality of life (see Figure 4). This situation was addressed by Kraemer et al. [44], who argued that the definition of a mediator does not necessarily imply the existence of treatment main effects, but that an interactive effect is sufficient. In the present study, treatment appears to have significantly influenced not only the *level *of the mediator (i.e., weight change) but also its *nature*, by "specifying" or creating the conditions under which it influenced a specific outcome (quality of life), even in the absence of differences between intervention and comparison groups for change in this variable.

According to Fox's physical self-perception model [45], global self-esteem stands hierarchically higher than body-esteem constructs. Therefore, we expected that the associations between changes in body image and weight might be paralleled in the results for self-esteem. However, only weight change affected self-esteem, without support for the reciprocal hypothesis (weight change being mediated by self-esteem change). This result replicates findings recently presented in a meta-analysis of well-being outcomes in weight loss treatments [11]. In that review, Blaine and colleagues proposed that weight loss influences self-esteem responses to treatment because significant reductions in weight prompt participants to internalize the more positive body-related appraisals they imagine others to have of them. In the present study, self-esteem was significantly associated with body shape concerns and with quality of life (but not with body size dissatisfaction), variables showing the same pattern of association with weight.

We observed that depressive symptoms improved in both intervention and comparison groups and were not a mechanism that influenced weight loss, nor were improvements in depression mediated by weight changes. These findings partially support previous reports where depression was associated with treatment (not the case in the present study), but not to weight loss. It is worth mentioning that the comparison group in our analysis did participate in a health education program. It involved less intervention contact and did not focus on weight but it might, nevertheless, have influenced participants' well-being, namely by social interaction with their group, by the continued contact with health professionals, and by some of the topics covered within the health education program (e.g., stress and time management).

## Conclusion

The primary concept under scrutiny in the current report was that treatment-related weight change and changes in selected psychological processes reciprocally affect each other during treatment. We tested this "reciprocal mediation" model for several variables usually considered as outcomes of weight loss programs, and found confirmation of our hypotheses for body image, especially body size dissatisfaction. Contrarily, the present results indicate that obesity-specific quality of life and self-esteem were affected *by *weight loss success but without reciprocal influences *back on *weight loss. To the extent psychosocial variables prove to be more than outcomes, as appears to be the case for body image, then the inclusion of contents to specifically change them during obesity treatment is clearly warranted [46]. We suggest that future studies follow this reciprocal mediation analysis procedure in randomized controlled trials with longer time periods, preferably with more than the two data points we have included in the present analysis, and include other variables such as motivation-related constructs (e.g., intrinsic motivation) or more classical social-cognitive variables (e.g, attitudes, perceived behavioral control). The inclusion of variables which are potentially moderating the effects of treatment (e.g number of previous diets, autonomous orientation [6]) might also be considered. The mediation and reciprocal effects analysis could be more elucidating in a study with these characteristics, thus providing more information about the dynamical mechanisms underlying weight management and help researchers improve the contents and conditions of obesity treatments.

## Competing interests

The authors declare that they have no competing interests.

## Authors' contributions

ALP and PJT conceived the study and drafted the manuscript. ALP performed the statistical analysis, was responsible for psychometric assessments and participated in the intervention's implementation. DAM participated in the study's conception and was the statistical advisor. MNS, PNV, TLB, SSM, CSM, and JTB actively participated in the intervention's implementation and in data collection. SOS participated in the study design and in the selection of psychosocial predictors. LBS is a principal investigator in the research trial. All authors read and approved the final manuscript.
